# Health Literacy and Unmet Needs in Patients With Multiple Sclerosis: A Cross‐Sectional Study

**DOI:** 10.1111/jocn.70396

**Published:** 2026-06-14

**Authors:** Francesco Pastore, Barbara Forastefano, Valentina Simonetti, Tommaso Guerra, Giancarlo Cicolini, Pietro Iaffaldano, Dania Comparcini

**Affiliations:** ^1^ Department of Precision and Regenerative Medicine and Jonian Area (DiMePRe‐J) Aldo Moro University of Bari Bari Italy; ^2^ Center of Innovation in Nursing Research (CINR)—Center for Advanced Studies and Technology (CAST) “G. D'annunzio” University of Chieti‐Pescara Chieti Italy; ^3^ Department of Biomedicine and Prevention TorVergata University Rome Italy; ^4^ Department of Innovative Technologies in Medicine & Dentistry “G.D'annunzio” University of Chieti‐Pescara Chieti Italy; ^5^ Neurology Unit, AOU Consorziale Policlinico Aldo Moro University of Bari Bari Italy; ^6^ DiBraiN—Department of Translational Biomedicine and Neuroscience Aldo Moro University of Bari Bari Italy; ^7^ Interdisciplinary Department of Medicine Aldo Moro University of Bari Bari Italy

**Keywords:** health literacy, informational needs, multiple sclerosis, needs assessment, unmet needs

## Abstract

**Aims:**

To identify a correlation between unmet needs and HL levels in people with Multiple Sclerosis (pwMS) and to evaluate how sociodemographic characteristics influence HL levels and unmet needs.

**Design:**

A cross‐sectional study.

**Methods:**

The study was conducted using a questionnaire including the HLS_19_‐Q12 to assess HL and the Long‐term Unmet Needs in Multiple Sclerosis tool, which evaluates five domains (neuropsychological, ambulation, physical, interpersonal relationship, and informational) and identifies whether needs are met or unmet and the desire for support.

**Results:**

Among the 116 participants included in the study, the overall HL level was sufficient. Mean scores across unmet needs domains were comparable. A significant difference in HL emerged in the informational domain, where participants reporting informational needs and a desire for support showed higher mean ranks. Although not significant, participants who acknowledged a need and expressed a desire for help showed higher mean ranks in HLS_19_‐Q12 scores across several domains. No significant correlations were found between HL and unmet needs domains.

**Conclusion:**

HL levels may enhance patients' ability to recognize and express needs without necessarily ensuring that these needs are met.

**Implications for the Profession and/or Patient Care:**

Routine assessment of both HL and unmet needs may help healthcare professionals identify patients who recognize problems but lack the structural support to address them.

**Impact:**

The impact of HL on need recognition and communication, together with the complexity and interconnectedness of unmet needs, highlights the need for healthcare systems to implement organizational, systemic, and multidimensional interventions aimed at promoting HL and effectively addressing patients' needs. Such strategies may support better disease management and improve quality of life in pwMS.

**Reporting Method:**

This study was reported according to STROBE checklist.

**Patient or Public Contribution:**

None.

## What Does This Paper Contribute to the Wider Global Clinical Community?

- This study highlights the role of health literacy (HL) in relation to unmet needs among people with multiple sclerosis (pwMS), showing how HL influences the ability to recognize, articulate, and seek help for these needs.
- The results provide insights for practice and policy by emphasizing the need for organizational and systemic HL actions, as well as a personalized, multidisciplinary approach to effectively address the complex needs of pwMS.

## 1 Introduction

The variability in the way Multiple Sclerosis (MS) manifests and progresses often contributes to the inability of people with MS (pwMS) to meet multiple needs (Maida et al. 2024) that go beyond symptoms directly related to the disease (Maida et al. 2024; Mohamed Abu Baker et al. 2023). In this context, the so‐called unmet needs emerge concerning various aspects, including financial support, personal fulfilment at a social and intellectual level, education, healthcare services, care, psychological support, and access to information (Florio‐Smith et al. 2023; Maguire et al. 2023; Mohamed Abu Baker et al. 2023; Pétrin et al. 2020).

Evidence shows that pwMS experience unmet needs, particularly in access to healthcare, social support, and assistance with daily living, highlighting potential systemic gaps in the management of MS within the healthcare context (Maguire et al. 2023; Maida et al. 2024; Pétrin et al. 2020).

Factors such as older age, low educational level, greater disability, and longer disease duration were associated with a higher number of unmet needs (Maida et al. 2024; McCabe et al. 2015). However, these sociodemographic and clinical factors do not fully explain the heterogeneity of unmet needs, as some patients report specific unmet needs regardless of motor disability or disease severity (Maida et al. 2024; McCabe et al. 2015), suggesting that these variables only partially account for their prevalence.

## 2 Background

One factor that enables individuals to shape their needs and the way they acquire and use health‐related information is Health Literacy (HL) (Liu et al. 2020). HL is defined as “an individual's ability to obtain and translate knowledge and information in order to maintain and improve health in a manner appropriate to individual and systemic contexts” (Liu et al. 2020). HL is therefore not limited to the knowledge, processing, and use of information related to health, healthcare, and healthcare systems, but also encompasses the ability to actively manage one's own health through self‐management practices (Liu et al. 2020). In this regard, HL plays a particularly relevant role in the context of chronic diseases (Larsen et al. 2022), in which individuals are required to actively participate in the daily management of their condition. An adequate level of HL indeed promotes active involvement in self‐care processes (Magi et al. 2026) and the adoption of appropriate health‐related behaviours (Elkin 2024). Higher levels of HL have been associated with more effective self‐care behaviours, including medication adherence, symptom monitoring, and the adoption of lifestyle changes (Magi et al. 2026), as well as with a better quality of life (QoL) (Zheng et al. 2018). Furthermore, across several chronic conditions, HL contributes to promoting self‐care‐oriented behaviours by increasing individuals' confidence and perceived control over health‐related decisions (Magi et al. 2026).

Individuals with a high HL level, in fact, can recognize when and where to find information, compare and understand it, and assess its reliability and relevance in relation to their own situation (Liu et al. 2020). These skills allow the individual to share such information with others, communicate their preferences effectively and clearly, and apply it in both individual and social decision‐making processes to achieve tangible benefits (Liu et al. 2020).

Evidence suggests a link between lower HL levels and the presence of unmet needs. In the general population, lower HL levels have been associated with a higher likelihood of developing unmet healthcare needs (Ko et al. 2025). Similarly, among breast cancer patients, lower HL levels have been associated with greater unmet informational needs (Halbach et al. 2016). A consistent finding has also been reported in the elderly Chinese population, where HL is associated with concern about unmet medical care needs (Li et al. 2024).

Among pwMS, lower HL is known to be associated with adverse health behaviours (increased risk of smoking, overweight, and obesity), greater use of healthcare services (emergency department visits and hospitalisations) (Baghini and Bahaadinbeigy 2023; Marrie et al. 2014), higher levels of stress (Dehghani 2024), increased symptom severity, and poorer QoL (Baghini and Bahaadinbeigy 2023; Claflin et al. 2021). Therefore, in this population, HL has been identified as a relevant mediating factor influencing self‐care, health promotion, medication adherence, use of healthcare services, and health outcomes (Baghini and Bahaadinbeigy 2023; Claflin et al. 2021; Dehghani 2021). However, to our knowledge, no study has yet examined the relationship between HL levels and unmet needs in pwMS. Exploring this relationship could be particularly relevant for guiding the development of communication and care strategies that help patients recognize, express, and meet their needs, which in turn may improve their participation in disease management (Members of the MS in the 21st Century Steering Group et al. 2018) and overall QoL (Katsarou et al. 2023).

## 3 The Study

### 3.1 Aim

This study aimed to identify a correlation between unmet needs and HL levels in pwMS and to evaluate how sociodemographic characteristics influence HL levels and unmet needs.

## 4 Methods

### 4.1 Design

A monocentre, observational, cross‐sectional study was conducted (February 2024–March 2025). The Strengthening the Reporting of Observational Studies in Epidemiology (STROBE) guidelines were used for accurate reporting of the research (von Elm et al. 2008).

### 4.2 Participants, Sampling and Setting

This study was conducted at an MS Centre, in southern Italy (Bari). A previously trained researcher was responsible for recruiting participants during their routine visits to the centre. Patients were recruited using a purposive sampling strategy, ensuring that all participants met the predetermined inclusion criteria.

### 4.3 Inclusion and Exclusion Criteria

Patients of both sexes, aged 18 years or older with a confirmed diagnosis of MS, included in the Italian Multiple Sclerosis and related Disorders Registry (RISM) (Trojano et al. 2019) database of the MS Centre, able to read and understand Italian, provide written informed consent, and without intellectual disabilities, severe psychiatric disorders, or cognitive impairment that could interfere with study participation were included. Patients who do not meet the inclusion criteria were excluded.

### 4.4 Data Collection Procedures, Instruments and Ethical Considerations

All procedures were conducted in accordance with the principles of the Declaration of Helsinki (World Medical Association 2025) and national and European data protection regulations. RISM was approved by the Ethics Committee of Policlinico di Bari (Study number: 55587/2016, Registro SM 001). Participation was voluntary. Patients signed an informed consent that allows the use of clinical data for research purposes.

Sociodemographic data (sex, age, education level) and clinical data (age at disease onset, diagnosed MS subtype, Expanded Disability Status Scale [EDSS] scores, follow‐up from first to last visit, latency from disease onset to diagnosis, latency from diagnosis to initiation of first Disease Modifying Therapy [DMT], age at initiation of first DMT, and current DMTs) were extracted from RISM‐app (Trojano et al. 2019). The additional variables were measured using a self‐reported questionnaire. During routine visits, eligible patients received oral explanations and an information sheet outlining the data collection procedures and the data anonymization process, provided by a nurse researcher. Patients who provided informed consent were personally given a questionnaire bearing an anonymous identification number to be completed immediately after the visit and returned in a sealed envelope. Personal identifiers were handled exclusively by the researcher to prevent any linkage with survey responses. All questionnaires were subsequently entered into a secure digital platform using a dedicated data entry form.

The questionnaire consisted of two sections aimed at assessing:

- *HL*: assessed using the validated Italian version (ISS 2020) of the HLS_19_‐Q12 instrument (Pelikan et al. 2022). This scale, developed within “The European Health Literacy Population Survey 2019‐2021 (HLS19)” of Measuring Population and Organizational Health Literacy (M‐POHL) (The HLS19 Consortium of the WHO Action Network M‐POHL 2021), includes 12 items rated on a 4‐point Likert scale (1 = very difficult; 4 = very easy). The overall HLS_19_‐Q12 score was calculated as the percentage (range 0–100) of items rated “easy” or “very easy,” provided that at least 80% of the items are completed. Based on score thresholds, HL levels are categorized as: Inadequate (≤ 50), Problematic (> 50 and ≤ 66.67), Sufficient (> 66.67 and ≤ 83.33) and Excellent (> 83.33) (Pelikan et al. 2022). In this study, the HLS_19_‐Q12 showed excellent internal consistency according to McDonald's *ω* (*ω* = 0.909).
- *Unmet Needs*: assessed using the Long‐term Unmet Needs in Multiple Sclerosis (LUN‐MS) tool (Mohamed Abu Baker et al. 2023). The LUN‐MS is a self‐administered questionnaire consisting of 29 items rated on a 3‐point Likert scale: 1 = No, I don't currently have this problem; 2 = Yes, I have this problem, but I don't need help with it; 3 = Yes, this is a problem and I would like help. The scale evaluates five domains of need: (i) neuropsychological (4 items), (ii) ambulation (4 items), (iii) physical (9 items), (iv) interpersonal relationship (4 items), and (v) informational (8 items). The score for each domain was calculated as the mean (range 1–3). The items are designed to identify whether these needs are met or unmet in pwMS and whether there is a desire to receive help in addressing them (Mohamed Abu Baker et al. 2023).

The scales were authorized for use by the authors (Mohamed Abu Baker et al. 2023; The HLS19 Consortium of the WHO Action Network M‐POHL 2021).

#### 4.4.1 Cross‐Cultural Adaptation and Validation of the LUN‐MS in an Italian Population

The LUN‐MS, originally developed in English, was translated into Italian using a rigorous forward–backward translation procedure in accordance with the methodological recommendations of Maneesriwongul and Dixon (2004). Two independent bilingual translators performed the forward translation from English to Italian, and a separate bilingual translator, blinded to the original version, conducted the backward translation into English. Discrepancies were discussed and resolved by consensus to ensure semantic and conceptual equivalence.

Content validity was subsequently assessed by a panel of six independent experts with experience in MS and psychometrics, who were not involved in the translation process. The experts independently evaluated the semantic, idiomatic, experiential, and conceptual equivalence of the original English and Italian versions. Their agreement supported the content validity of the Italian version. No cultural adaptation, item modification, or deletion was required. The pre‐final Italian version was pilot‐tested in a sample of 25 pwMS who were not involved in the present study. The pilot testing confirmed the clarity, comprehensibility, and acceptability of all items, and no further revisions were deemed necessary. The original five‐domain structure of the instrument was retained to ensure conceptual consistency with the source version. Internal consistency was assessed for each of the five theoretical domains. Reliability indices indicated good to excellent internal consistency (McDonald's *ω* = 0.76–0.93), supporting the use of subscale scores for subsequent analyses.

### 4.5 Sample Size Estimation

The sample size calculation was based on the planned data analyses (Spearman's *ρ*), with a *p*‐value of 0.05, a power of 0.80, and a medium effect size (*ρ* = 0.3), which indicated a required minimum of 84 participants.

### 4.6 Data Analysis

Analyses were performed using IBM SPSS 28.0 Statistics (IBM Corp), with a statistical significance level set at a *p*‐value < 0.05. Participants with incomplete registry data were excluded from the analyses. Overall, 134 subjects were initially screened, and 18 were excluded because of missing data in the registry dataset. Means and standard deviations (SD) and medians and interquartile ranges (IQR) were calculated for continuous variables, while categorical variables were reported through frequencies and percentages.

Differences in HL scores, on a 0–100 scale, in relation to unmet needs across the five domains and sociodemographic variables, as well as the differences between unmet needs in the five domains and sociodemographic variables, were explored using the Kruskal–Wallis test and the Mann–Whitney *U* test. To explore the correlation between the HL score and the LUN‐MS domains, Spearman's correlation analysis was conducted.

## 5 Results

### 5.1 Characteristics of the Sample

A total of 116 individuals participated in the study. Sociodemographic and clinical characteristics are summarized in Table 1.

**TABLE 1:** Sociodemographic and clinical characteristics (*N* = 116).

Characteristics	
**Sex, *n* (%)**	
Female	85 (73.3)
Male	31 (26.7)
**Age (years), mean (SD)**	44.2 (11.9)
**Educational level, *n* (%)**	
Primary school	1 (0.9)
Lower secondary school	28 (24.1)
Higher secondary school	53 (45.7)
Bachelor/higher	34 (29.3)
**Age at disease onset (years), mean (SD)**	28.8 (9.4)
**Diagnosed MS subtype, *n* (%)**	
RRMS	111 (95.7)
PPMS	1 (0.9)
SPMS	4 (3.4)
**EDSS, median (IQR)**	2 (0–5)
**Follow up from first to last visit (years), median (IQR)**	11.6 (7.4–21.6)
**Time from disease onset to diagnosis (years), median (IQR)**	1.7 (0.4–4.9)
**Time from diagnosis to initiation of the first DMT (years), median (IQR)**	0.3 (0.1–0.7)
**Age at initiation of the first DMT (years), mean (SD)**	32.5 (10.4)
**Ongoing DMT(s), *n* (%)**	
Interferon	65 (56)
Dimethyl fumarate	17 (14.7)
Natalizumab	9 (7.8)
Glatiramer acetate	8 (6.9)
Cladribine	8 (6.9)
Other DMT(s) (Teriflunomide, Fingolimod, Ocrelizumab, Rituximab, and Ofatumumab)	37 (32.7)

Abbreviations: DMT, disease modifying therapy; EDSS, Expanded Disability Status Scale; MS, multiple sclerosis; PPMS, primary progressive multiple sclerosis; RRMS, relapsing remitting multiple sclerosis; SPMS, secondary progressive multiple sclerosis.

### 5.2 HL and Comparison of HLS_19_ ‐Q12 Scores by Sex, Age Groups, and Educational Level

The overall HLS_19_‐Q12 mean score was 72 (SD = 24.2). Participants' HL levels were categorized as Excellent (33.6%; *n* = 39), Sufficient (31%; *n* = 36), Inadequate (23.3%; *n* = 27), and Problematic (12.1%; *n* = 14).

Regarding the comparison between education level and HLS_19_‐Q12 scores, no statistically significant difference was observed (*H* = 4.553; *p* = 0.208). Furthermore, the results did not indicate any statistically significant differences between males and females or across age groups (Table 2).

**TABLE 2:** Comparison of HLS_19_‐Q12 scores by gender, age groups, and educational level.

Sociodemographic characteristics	*n*	Mean rank of HLS_19_‐Q12 scores	Kruskal–Wallis *H*/Mann–Whitney *U*	*p*
**Sex**				
Female	85	56.2	1125.500	0.226
Male	31	64.7		
**Age groups (years)**				
< 30	13	58.6	6.203	0.184
30–39	29	68.9		
40–49	35	60.5		
50–59	28	50.0		
≥ 60	11	46.4		
**Educational level**				
Primary school	1	1.0	4.553	0.208
Lower secondary school	28	62.8		
Higher secondary school	53	60.8		
Bachelor or higher	34	53.2		

### 5.3 Unmet Needs and Differences in the Expression of Needs Across Five Domains According to Age Groups and Education Levels

Mean scores across domains were as follows: neuropsychological 2 (SD = 0.7), ambulation 2 (SD = 0.9), physical 2 (SD = 0.7), interpersonal relationships 2 (SD = 0.8), and informational 2 (SD = 0.6). The percentage of responses for each item is reported in Table 3.

**TABLE 3:** Percentage of responses for each item across the five LUN‐MS domains.

Domains/Items	Answers		
	No, I don't currently have this problem, *n* (%)	Yes, I have this problem, but I don't need help with it, *n* (%)	Yes, this is a problem, and I would like help, *n* (%)
**Neuropsychological**			
21	46 (39.7)	28 (24.1)	42 (36.2)
22	50 (43.1)	19 (16.4)	47 (40.5)
23	45 (38.8)	29 (25)	42 (36.2)
24	51 (44)	22 (19)	43 (37.1)
**Ambulation**			
6	54 (46.6)	13 (11.2)	49 (42.2)
7	51 (44)	8 (6.9)	57 (49.1)
18	53 (45.7)	6 (5.2)	57 (49.1)
19	51 (44)	7 (6)	58 (50)
**Physical**			
8	51 (44)	6 (5.2)	59 (50.9)
9	45 (38.8)	18 (15.5)	53 (45.7)
10	51 (44)	6 (5.2)	59 (50.9)
11	52 (44.8)	10 (8.6)	54 (46.6)
12	47 (40.5)	17 (14.7)	52 (44.8)
13	53 (45.7)	10 (8.6)	53 (45.7)
14	53 (45.7)	17 (14.7)	46 (39.7)
15	17 (14.7)	19 (16.4)	51 (44)
16	51 (44)	15 (12.9)	50 (43.1)
**Interpersonal relationship**			
26	52 (44.8)	11 (9.5)	53 (45.7)
27	52 (44.8)	10 (8.6)	54 (46.6)
28	52 (44.8)	9 (7.8)	55 (47.4)
29	49 (42.2)	11 (9.5)	56 (48.3)
**Informational**			
1	54 (46.6)	22 (19)	40 (34.5)
2	54 (46.6)	17 (14.7)	45 (38.8)
3	50 (43.1)	22 (19)	44 (37.9)
4	49 (42.2)	14 (12.1)	53 (45.7)
5	50 (43.1)	19 (16.4)	47 (40.5)
17	50 (43.1)	11 (9.5)	55 (47.4)
20	51 (44)	17 (14.7)	48 (41.4)
25	42 (36.2)	21 (18.1)	53 (45.7)

Regarding education levels, the analysis revealed a significant difference in the domain of neuropsychological needs (*p* = 0.008), with participants holding a bachelor's degree or higher showing a higher mean rank compared to those with lower education levels (Figure 1). Participants with a bachelor's degree or higher also showed higher mean ranks in the domains of ambulation, physical, interpersonal relationships, and informational. However, these differences were not statistically significant (*p* > 0.05). No statistically significant differences were observed between age groups (*p* > 0.05) (Table 4).

**FIGURE 1:**
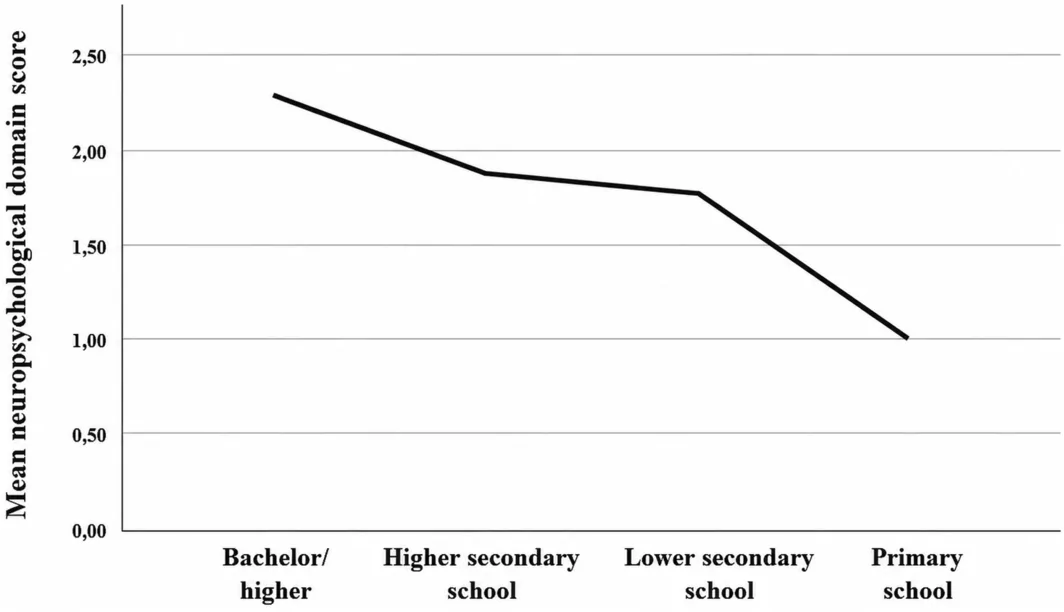
Mean neuropsychological unmet needs scores across educational levels.

**TABLE 4:** Differences in the expression of needs across five domains according to age groups and education levels.

LUN‐MS domains/Subgroup	Neuropsychological			Ambulation			Physical			Interpersonal relationship			Informational		
	Mean rank	*H*	*p*	Mean rank	*H*	*p*	Mean rank	*H*	*p*	Mean rank	*H*	*p*	Mean rank	*H*	*p*
**Age groups (years)**															
< 30	54.9	2.656	0.617	60.8	1.542	0.819	61.9	1.780	0.776	63.4	3.606	0.462	55.6	1.280	0.865
30–39	52.6			60.2			55.6			55.7			55.1		
40–49	58.5			52.9			54.3			53.3			57.0		
50–59	66.5			61.0			62.5			60.0			63.4		
≥ 60	58			62.7			65.6			73.0			63.2		
**Educational level**															
Primary school	11.5	11.935	**0.008**	19.5	3.449	0.327	9.5	4.320	0.229	17	2.257	0.521	2.5	5.888	0.117
Lower secondary school	50.0			51.5			51.4			54.6			53.7		
Higher secondary school	54.1			60.6			59.8			59.9			56.5		
Bachelor or higher	73.8			62.2			63.7			60.8			67.3		

Abbreviation: *H*, Kruskal–Wallis test.

### 5.4 Differences in Mean Ranks of HLS_19_ ‐Q12 Scores in Relation to the Expression of Unmet Needs Across the Five LUN‐MS Domains

Differences in mean ranks of HLS19‐Q12 scores were analysed across all three groups based on the expression of unmet needs and the desire for support within each LUN‐MS domain, using the Kruskal–Wallis test. The analysis revealed a statistically significant difference only in the informational domain (*H* = 11.787; *p* = 0.003), in which participants experiencing an informational problem and indicating a need for help had a significantly higher mean rank than those who did not report the problem or did not require support. No statistically significant differences were found in the neuropsychological (*H* = 2.857; *p* = 0.240), ambulation (*H* = 1.821; *p* = 0.402), physical (*H* = 3.360; *p* = 0.186), and interpersonal relationship (*H* = 1.914; *p* = 0.384) domains. A general pattern indicated that participants who acknowledged a need and expressed a desire for help showed higher mean ranks in HLS_19_‐Q12 scores within the ambulation, physical, and interpersonal relationship domains, compared with the other two groups (Table 5).

**TABLE 5:** Differences in mean ranks of HLS_19_‐Q12 scores in relation to the expression of unmet needs across the five LUN‐MS domains (*N* = 116).

LUN‐MS domains	Groups	*n*	Mean rank of HLS_19_‐Q12 score	Kruskal–Wallis *H*	*p*
**Neuropsychological**	No, I don't currently have this problem	57	54.5	2.857	0.240
	Yes, I have this problem, but I don't need help with it	38	66		
	Yes, this is a problem, and I would like help	21	55.9		
**Ambulation**	No, I don't currently have this problem	52	56.2	1.821	0.402
	Yes, I have this problem, but I don't need help with it	24	54.1		
	Yes, this is a problem, and I would like help	40	64.2		
**Physical**	No, I don't currently have this problem	50	55.5	3.360	0.186
	Yes, I have this problem, but I don't need help with it	53	57.5		
	Yes, this is a problem, and I would like help	13	74.2		
**Interpersonal relationship**	No, I don't currently have this problem	52	57.8	1.914	0.384
	Yes, I have this problem, but I don't need help with it	32	53.4		
	Yes, this is a problem, and I would like help	32	64.8		
**Informational**	No, I don't currently have this problem	54	54.9	11.787	**0.003**
	Yes, I have this problem, but I don't need help with it	55	56.8		
	Yes, this is a problem, and I would like help	7	100.2		

### 5.5 The Relationships Between the HLS_19_ ‐Q12 Score and the Five Domains of the LUN‐MS

The analysis revealed all statistically significant inter‐domain correlations for LUN‐MS (*p* ≤ 0.001). In particular, the informational domain was strongly correlated with the physical (*ρ* = 0.612) and neuropsychological (*ρ* = 0.653) domains, while the ambulation domain was highly correlated with the physical (*ρ* = 0.872) and interpersonal relationship (*ρ* = 0.764) domains. A strong correlation was also found between the physical and interpersonal relationship domains (*ρ* = 0.773). However, no statistically significant correlations emerged between the HLS_19_‐Q12 score and any of the five domains: neuropsychological (*ρ* = 0.101; *p* = 0.279), ambulation (*ρ* = 0.051; *p* = 0.588), physical (*ρ* = 0.130; *p* = 0.166), interpersonal relationship (*ρ* = 0.054; *p* = 0.562), and informational (*ρ* = 0.143; *p* = 0.125) (Figure 2).

**FIGURE 2:**
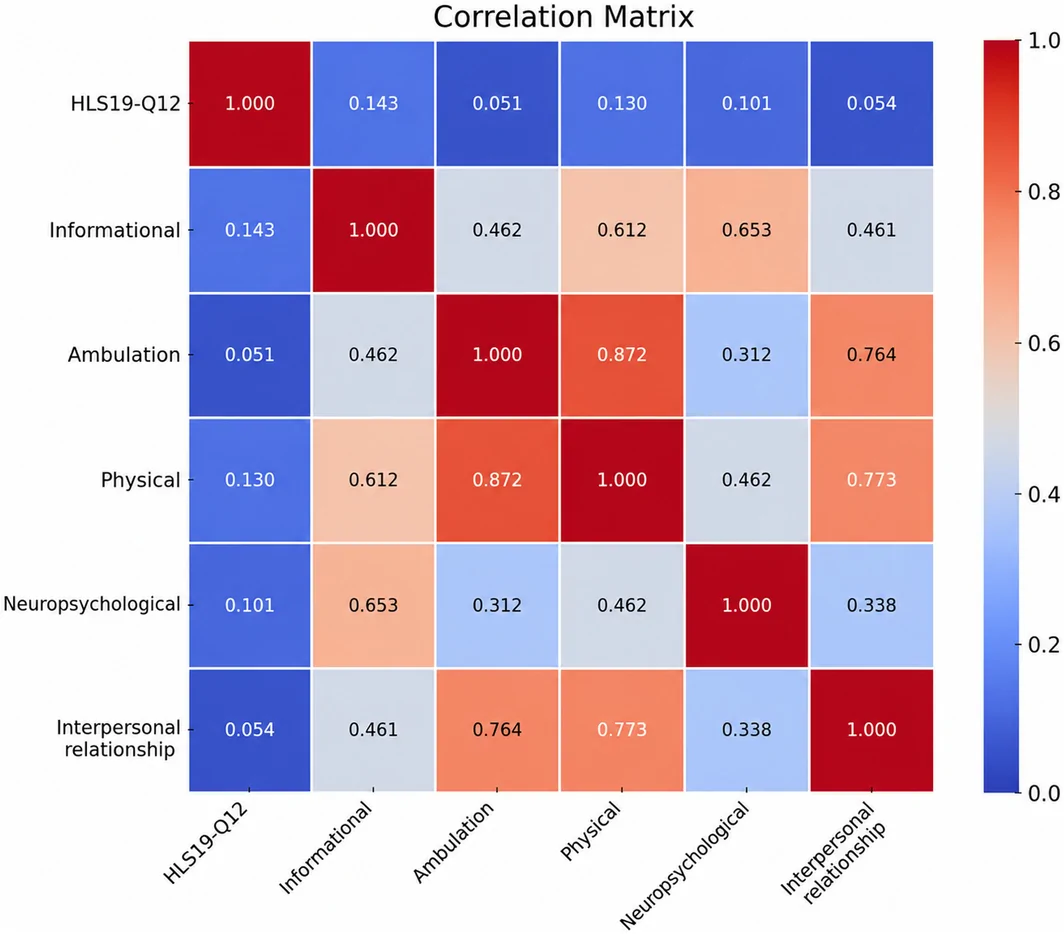
Correlation matrix between the HLS_19_‐Q12 score and the five domains of the LUN‐MS. [Colour figure can be viewed at wileyonlinelibrary.com]

## 6 Discussion

The study explored the relationship between unmet needs and HL levels in pwMS and examined the influence of sociodemographic characteristics on both HL levels and unmet needs.

The overall HL level among pwMS was sufficient, confirming the findings of a recent review (Baghini and Bahaadinbeigy 2023), which highlights adequate or acceptable HL levels in pwMS. However, one‐third of participants reported inadequate or problematic HL levels. This suggests significant disparities in the ability to access, understand, and use health information within this population.

Comparison of HLS_19_‐Q12 scores by sex showed no statistically significant differences. However, male participants reported higher HL scores compared to females, consistent with findings in chronic illnesses populations (Sun et al. 2022). Although our findings do not allow a conclusive interpretation of these differences, the evidence suggests that sex and gender should be considered potential factors contributing to disparities in HL (Bonaccorsi et al. 2023). Such disparities may reflect broader socio‐cultural and behavioural factors, including differences in health‐seeking behaviours, caregiving roles (Shin and Baek 2025), online health information‐seeking behaviours, and digital HL (Bonaccorsi et al. 2023). Although further studies are needed to clarify the mechanisms underlying these differences in pwMS, these observations highlight the need for sex‐sensitive educational approaches aimed at improving HL also in this population of patients.

In our sample, HL scores tended to decrease with increasing age, although these differences were not statistically significant. In pwMS, cognitive decline may occur over time, which could reduce an individual's ability to understand and manage health‐related information, and thus their HL levels (Marrie et al. 2014). Therefore, considering that cognitive abilities mediate the relationship between HL and self‐care, interventions aimed at improving HL should be adapted to patients' different cognitive abilities, particularly among older individuals (Magi et al. 2026).

Unlike HL, for which comparison of HLS_19_‐Q12 scores according to educational level showed no statistically significant differences, regarding unmet needs, participants with a bachelor's degree or higher reported significantly higher scores in the neuropsychological domain than those with lower educational levels. Patients with a higher educational level also reported higher scores in all other domains, although these differences were not statistically significant. This finding is consistent with previous studies (Lee et al. 2020; Yang et al. 2019) and suggests that a higher education may influence the ability to recognize and express these needs. Individuals with higher education levels may have higher expectations for well‐being (Kristoffersen 2018); and may perceive certain aspects as needs that individuals with lower educational levels may not consider relevant or necessary. Indeed, education fosters desirable outcomes and promotes a sense of personal efficacy, which supports autonomy in pursuing important goals, including maintaining health (Mirowsky and Ross 2005).

However, the interpretation of these findings requires further consideration from a broader perspective. Indeed, education, HL, and cognitive abilities are closely interconnected. Education appears to be one of the major personal determinants of HL (Sørensen et al. 2012; Stormacq et al. 2019), yet individuals with the same educational level may present different HL levels and cognitive abilities (Stormacq et al. 2019). Education may not be directly associated with HL, as the latter may be influenced by other social, environmental, and situational determinants (Sørensen et al. 2012). Similarly, educational attainment, due to differences in educational experiences and variability in educational pathways and contexts, may not automatically reflect an individual's cognitive abilities (Stormacq et al. 2019). Therefore, educational level may act both as an independent determinant and as an indicator of broader competencies, including cognitive abilities as well as opportunities to access resources. In light of these considerations, further research is warranted, as the observed findings may not exclusively reflect the effect of education itself, but also the indirect influence of cognitive abilities. In this sense, education, HL, and cognitive resources may jointly contribute to enhancing the ability to recognize, interpret, and express one's health needs.

Indeed, in the informational domain, which includes needs related to disease understanding, treatment, support services, and daily management, this study showed that patients with higher HL scores demonstrated a greater ability to recognize, express, and seek help. A similar trend was observed across other domains: patients who expressed a need and sought support tended to have higher HL levels than those who did not recognize the problem or did not wish to seek help.

Importantly, overall HL was not directly correlated with unmet needs across the five domains, indicating that HL alone does not automatically reduce unmet needs. Although HL may facilitate the recognition of one's needs, the ability to express them, and help‐seeking behaviours, their actual fulfilment is likely also influenced by structural and organizational factors. Indeed, HL does not operate independently but is shaped by social and environmental contexts (Magi et al. 2026). This finding challenges a simplified view of HL as a direct determinant of unmet needs. In chronic conditions, in which HL represents a key factor associated with self‐care ability, its impact may be influenced by psychological, cognitive, and social variables (Magi et al. 2026). Factors such as emotional distress, motivation, cognitive deficits, socioeconomic barriers, the availability of external support mechanisms, support provided by healthcare professionals, as well as the level of complexity required for disease management may strengthen or weaken the role of HL in self‐care processes (Magi et al. 2026).

In the context of MS, patients often have complex needs that depend not only on HL skills, but also on disease severity, the availability of social and healthcare support, and individual background (Maida et al. 2024). Even patients with sufficient HL levels may struggle to address physical or mobility‐related needs without access to appropriate healthcare resources and support services (Maida et al. 2024). For instance, a patient may know how to manage fatigue but remain unable to cope without adequate support or effective pharmacological treatment.

Consistent with this complexity, all five domains were significantly correlated, suggesting that unmet needs tend to coexist and influence each other. This aligns with prior evidence showing that motor impairment in MS affects not only physical functioning but also emotional, neuropsychological, and social well‐being (Brandão et al. 2022). Walking difficulties and symptoms such as fatigue and pain reduce functional autonomy and negatively impact social participation, integration, and emotional health (Bass et al. 2020; Söylemez et al. 2024). Given that HL alone does not ensure need fulfilment, individual‐level interventions may be insufficient. Although MS nurses may represent a critical resource for improving HL, promoting timely help‐seeking behaviours, and supporting more effective self‐management practices (Pastore et al. 2026); our findings support a shift towards organizational and systemic strategies. Developing an organizational culture oriented towards HL is essential, with HL integrated into the organizational strategy by top management to improve patients' HL and quality of care (Bonaccorsi et al. 2020). Furthermore, a personalized and multidisciplinary approach, combining therapeutic, psychoeducational, and rehabilitative support, is crucial to address patients' complex needs effectively and enhance QoL (Ponzio et al. 2020). Evidence from Ireland demonstrates that community workers providing tailored support to pwMS, through structured physiotherapy programmes, symptom management initiatives, peer‐learning events, social support, and educational sessions, can effectively meet a wide range of patient needs. These interventions have been shown to meet informational, emotional, and care coordination needs, facilitating adaptation to living with MS (Maguire et al. 2023). Health policies should recognize and integrate such support models to better address the needs of pwMS.

### 6.1 Strengths, Limitations and Recommendations for Further Research

Although the study contributes to understanding the relationship between HL and unmet needs among pwMS, several limitations should be acknowledged. First, the cross‐sectional design of the study precludes any causal inference. Second, while the HLS_19_‐Q12 offers a valuable and standardized framework for HL assessment, future studies could benefit from the use of MS‐specific HL instruments. Such tools may offer greater sensitivity in capturing the disease‐related nuances of HL in pwMS, enabling a more tailored and comprehensive understanding of HL within this population. Furthermore, the sample was limited to patients from a single MS Centre, selected using purposive sampling, which may have introduced selection bias. These limitations may affect the generalizability of the findings to the broader MS patient population, highlighting the need for further longitudinal and multicentre research to better understand the nature of these relationships. Finally, future studies should include additional variables, such as disease severity and levels of social and healthcare support, to offer a more comprehensive understanding of the relationship between HL and unmet needs and clarify their combined effect on patients' outcomes.

### 6.2 Implications for Policy and Practice

Routine assessment of both HL and unmet needs may help healthcare professionals identify patients who recognize problems but lack structural support to address them, as well as those who do not recognize or are unable to express their needs.

Organizational strategies should promote a HL–responsive healthcare environment, capable of integrating both the assessment and enhancement of HL, helping patients recognize their needs and access appropriate care.

Nurses play a key role in assessing and responding to patients' needs. Therefore, it is essential that they adopt assessment tools, educational strategies, and HL‐sensitive approaches to facilitate patients' understanding and expression of their needs.

Since HL alone does not guarantee that needs are met, nursing practice should extend beyond patient education to include coordination of care within multidisciplinary teams to effectively respond to the needs of pwMS.

## 7 Conclusions

This study demonstrated that higher HL levels influence patients' ability to recognize and express specific needs, particularly informational ones, without necessarily ensuring that these needs are met. Unmet needs also tend to coexist and be interrelated. The impact of HL on need recognition and communication, together with the complexity and interconnectedness of unmet needs, highlights the need for healthcare systems to implement organizational, systemic, and multidimensional interventions aimed at promoting HL and effectively addressing patients' needs. Such strategies may support better disease management and improve QoL in pwMS.

## Author Contributions

**Francesco Pastore:** conceptualization, methodology, investigation, data curation, writing – original draft, writing – review and editing. **Barbara Forastefano:** conceptualization, methodology, data curation, writing – original draft, writing – review and editing. **Valentina Simonetti:** conceptualization, methodology, writing – original draft, validation, writing – review and editing. **Tommaso Guerra:** data curation, formal analysis, writing – review and editing. **Giancarlo Cicolini:** conceptualization, writing – original draft, visualization, writing – review and editing. **Pietro Iaffaldano:** conceptualization, investigation, writing – original draft, supervision, writing – review and editing. **Dania Comparcini:** conceptualization, methodology, writing – original draft, supervision, validation, writing – review and editing. All authors read and approved the final manuscript.

## Funding

The authors have nothing to report.

## Disclosure

Statistics: There is an expert statistician on the author team: Dr. Tommaso GUERRA, Neurology Unit, AOU Consorziale Policlinico, Aldo Moro University of Bari, Bari, Italy.

## Conflicts of Interest

The authors declare no conflicts of interest. P. Iaffaldano received grants to attend scientific congresses or speaker honoraria from Biogen, Merck‐Serono, Novartis, Roche, Sanofi/Genzyme, and Teva.

## Supporting information


**Data S1:** STROBE Statement—Checklist of items that should be included in reports of *cross‐sectional studies*.

## Data Availability

The data supporting this study's findings are available on request from the corresponding author.
